# Educational needs of patients, families, and healthcare professionals to support the patient journey in haemophilia gene therapy in the UK

**DOI:** 10.1186/s13023-023-02977-y

**Published:** 2023-11-25

**Authors:** Sara Boyce, Simon Fletcher, April Jones, Ruchika Kohli, Sarah Mangles, Min Ong, Debra Pollard, Sujan Sivasubramaniyam, David Stephensen, Nicola Stoner, Rashid Kazmi

**Affiliations:** 1 University Hospital Southampton, Southampton, UK; 2 Oxford University Hospitals NHS Foundation Trust, Oxford, UK; 3 The Royal Victoria Infirmary, Newcastle upon Tyne, UK; 4 CSL Behring, West Sussex, UK; 5 Haemophilia, Haemostasis and Thrombosis Centre, Hampshire Hospitals NHS Foundation Trust, Basingstoke, UK; 6 Sheffield Children’s NHS Foundation Trust, Sheffield, UK; 7 Haemophilia and Thrombosis Centre, Royal Free Hospital, London, UK; 8 East Kent Hospitals University NHS Foundation Trust, Kent, UK

**Keywords:** Gene therapy, Haemophilia A, Haemophilia B, Health services needs and demand, Patient navigation

## Abstract

With the first gene therapies for haemophilia approved by the European Commission, the US Food and Drug Administration, and the Medicines and Healthcare products Regulatory Agency, it is important to consider the remaining unmet needs and challenges that may arise throughout patients’ treatment journeys. We discuss existing unmet needs and important considerations prior to, during, and following haemophilia gene therapy treatment in the UK, and propose potential next steps. Key areas for attention are education, psychological support, and guidance on implementation. Strategies are urgently required to fulfil these needs. An immediate priority for information providers should be comprehensive education for people with haemophilia (PWH) and healthcare professionals (HCPs). Greater access to resources and training in psychological services will be required throughout the treatment pathway. More specific guidance is required to define the implementation model, criteria for accreditation, and responsibilities of care centres. Furthermore, PWH may revisit discussions with HCPs multiple times pre-infusion, thus the patient journey is unlikely to be linear. Consideration of these challenges, and of potential strategies to address them, will be integral to optimising the future success of this promising therapy.

To the Editor

The first gene therapy for haemophilia A was conditionally approved in 2022 by the European Medicines Agency (EMA) [1], with US Food and Drug Administration (FDA) approval granted in 2023 [2]. The first gene therapy for haemophilia B was approved in 2022 by the FDA [3]; following a positive EMA Committee for Medicinal Products for Human Use opinion reported in December 2022 [4, 5], the European Commission and the Medicines and Healthcare products Regulatory Agency granted further approvals for this therapy in early 2023 [6, 7]. Draft guidance published by the National Institute for Health and Care Excellence (NICE) in mid-2023 recommended against provision of the approved haemophilia B gene therapy by the National Health Service (NHS) in England, citing cost-effectiveness uncertainties; at the time of this letter’s publication, however, NICE is due to re-evaluate evidence following consultation with stakeholders in Q4 2023, and recommendations may be amended prior to final publication of the guidance [8]. While commissioning processes in England have begun and implementation of gene therapy for haemophilia in the UK seems imminent, the patient journey through gene therapy treatment pathways is new to many healthcare professionals (HCPs), who may have unmet educational requirements. We highlight important considerations, existing unmet needs, and potential challenges faced by people with haemophilia (PWH) throughout their gene therapy journey in the UK. This letter was developed based on our experiences as UK HCPs, and our conclusions are made with specific regard to gene therapy for haemophilia in the UK.

## Unmet educational needs

A key requirement is education about gene therapy for haemophilia, for PWH and their families, and for the multidisciplinary team (MDT) of HCPs. Patient education by HCPs has been predominantly verbal or within clinical trials thus far. Materials to help guide decisions on gene therapy have been produced by patient organisations such as the European Haemophilia Consortium (EHC) [9]. However, lack of understanding of available resources and their reliability may deter PWH from seeking gene therapy education. Guidance for PWH may be valuable, such as a centrally compiled list of easily accessible educational materials. National patient advocacy groups will be crucial in delivering patient education.

**Fig. 1:**
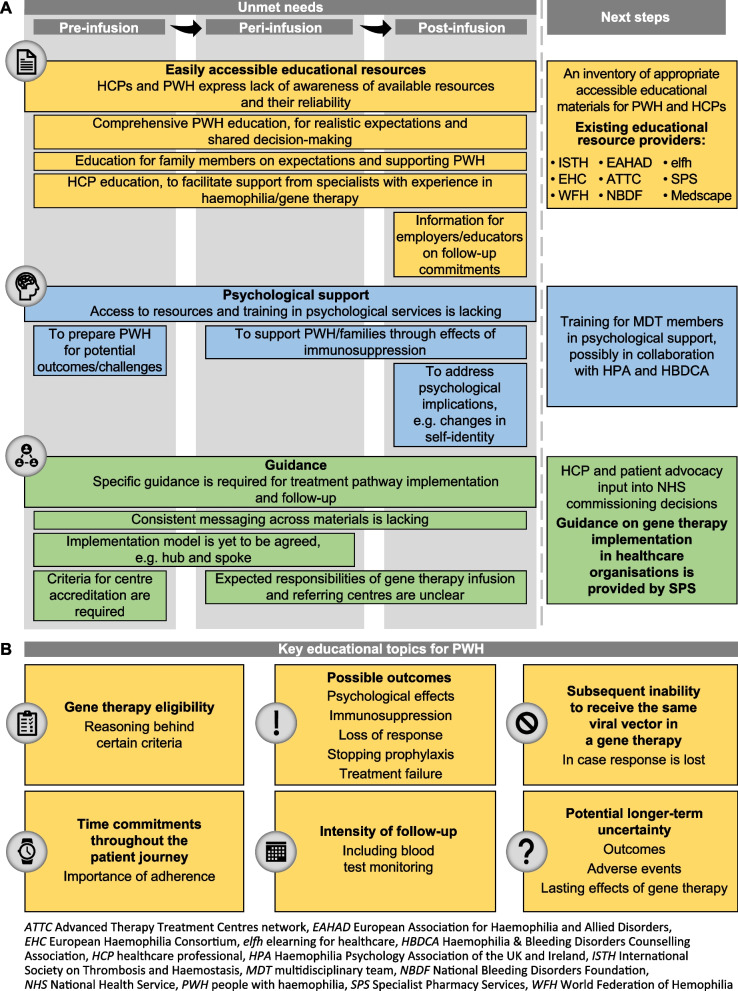
Unmet needs throughout the patient journey, potential next steps, and educational requirements for PWH. **A** Current unmet needs throughout the gene therapy for haemophilia journey and potential starting points regarding education, psychological support, and guidance. Time periods are indicated by a light grey background; unmet needs are applicable to all time periods across which they span. **B** Summary of key educational topics required for PWH. Education for PWH on these topics should begin as soon as possible pre-infusion, ideally in childhood

PWH usually seek information from HCPs at their current treatment centre. Materials for all MDT members will be necessary to ensure sufficient understanding to explain this complex topic to PWH, or signpost resources, the goal of which is to allow true shared decision-making. Materials exist that could facilitate HCP education, produced by groups such as those listed in Fig. 1A [10–16]. The World Federation of Hemophilia Shared Decision Making Tool is one such readily available resource [14]. Furthermore, the European Association for Haemophilia and Allied Disorders established a working group in 2019 to provide information to PWH and HCPs on practical and safety-related aspects of gene therapy [17]. Although special interest groups share such materials with members, widespread knowledge of resources is limited. An inventory of appropriate available resources could provide initial direction to HCPs.

HCP education should include a diverse set of relevant topics. Some of the essential topics requiring education, however, may not be apparent until gene therapy implementation, as topics and extent of education may depend on currently unknown factors, e.g. the responsibilities of different centres and long-term monitoring requirements. National specialist support platforms could help identify and meet specialists’ educational needs. The UK Haemophilia Centre Doctors’ Organisation (UKHCDO) and The Haemophilia Society could be instrumental in achieving this.

Consistent messaging across educational materials for PWH, their families, and HCPs is lacking but it will be important to manage expectations and avoid confusing misinformation. As echoed in the 2022 EHC conference, this could be achieved through collaborative working groups with input from academia, clinicians, PWH and their families, and industry. For example, in the gene therapy for spinal muscular atrophy (SMA) setting, the SMA UK network has been valuable in delivering education and facilitating consistency of resources. The Advanced Therapy Treatment Centres network [13] and Specialist Pharmacy Service (SPS) [18] resources have been achieved through such working groups. Due to the dynamic nature of the field, it will be important for educational materials to be continuously updated with further advances, as is the case with SPS resources.

## Pre-infusion considerations

PWH and their families should be fully informed pre-infusion to enable truly shared decision-making. Discussions should take place between the MDT, PWH, and families around whether gene therapy might align to the patient’s goals, the available alternative options, and informed preferences [19]. PWH may revisit discussions with HCPs multiple times pre-infusion before deciding on gene therapy. To ensure that PWH are suitably informed and minimise the risk of treatment remorse after receiving this irreversible treatment, comprehensive patient education on gene therapy is needed pre-infusion and should ideally begin in childhood/adolescence. Information should address concerns and manage expectations, and clinicians should check that PWH understand information, using teach-back techniques, for example. Key topics for patient education are summarised in Fig. 1B. Families of PWH considering gene therapy will require education on what to expect, as will employers and educators, given the time commitments of initial follow-up.

Prior to infusion, it is important to consider factors that affect eligibility for gene therapy. Case-by-case evaluation of PWH who meet eligibility criteria may further inform a patient’s decision, e.g. motivations, expectations, and commitments to adhere to required follow-up to maintain their safety. Alternative treatment options that may suit needs and lifestyle of PWH should be explored. Concern within the haemophilia community that potentially eligible patients may not have the opportunity to explore gene therapy as a treatment option because of selection bias should be mitigated. Some PWH will have no desire to receive gene therapy; patients’ decisions must be respected.

Equitable access to psychosocial professionals and hepatologists with experience in haemophilia care and gene therapy is needed for gene therapy implementation in the UK. Psychological support will help PWH and their families to process the decision, mentally prepare them for potential challenges, or help PWH deal with disappointment of exclusion (due to pre-existing viral vector antibodies or liver cirrhosis, for example). Baseline psychological assessment may help monitor the benefits or desirable/undesirable effects of the treatment. This may also help assess current gaps in patient education and patients’ activation measure [20], to determine patients’ engagement in their care. Patient motivations and engagement, however, may change with time.

## Peri-infusion considerations

In the UK, an MDT consisting of haematologists with an interest in haemophilia, nurses, psychologists, hepatologists, and pharmacists is needed to support PWH throughout the infusion process and manage potential side effects. This list of specialist team members is not exhaustive and will evolve as current knowledge on gene therapy and understanding of its effect on other organs advances. Furthermore, this stage may not involve the full extended MDT members who could be required pre- and post-infusion. Hepatologist involvement will be important for the management of transaminitis and to advise on suitability of concomitant medication. PWH undergoing gene therapy may wish for a family member/caregiver/partner to be present during the infusion. Thus, support from a carer or nurse will be beneficial for both families and PWH. Gene therapy training for all HCP groups should therefore be implemented prior to and in parallel with gene therapy availability.

## Post-infusion considerations

Post-infusion follow-up care is critical to ensure standardisation across different treatment centres. UK-specific guidance is currently being drafted by the UKHCDO, which undertakes research on, and produces guidelines for, management of inherited bleeding disorders [21]. Unmet needs regarding guidance on implementation and follow-up are summarised in Fig. 1A. Follow-up care guidance should ideally be standardised across the UK. For instance, close monitoring will likely be required for 12–24 h post-infusion, with follow-up of 10–15 years, regardless of region. In 2022, the International Society on Thrombosis and Haemostasis Scientific and Standardization Committee established a working group to oversee standardisation of gene therapy implementation [22]. Follow-up care resourcing within centres will need to be effectively managed. Although potentially less intense than in clinical trials, real-world patient follow-up will be regular and have significant impacts on centre resources, particularly on nursing or clinician time, depending on centres’ setup.

Psychologist support for both PWH and their families will be critical post-treatment given the potential psychological implications of gene therapy. PWH may struggle with adapting to new circumstances and experiences, with changes in their factor levels and disease burden status. Families may also be affected by the physical and emotional changes post-infusion. The effects of immunosuppression (often required to address increased liver enzyme levels) have been reported by PWH and family members as the most unpleasant aspect of receiving haemophilia gene therapy [23]. In addition to having associations with immune-mediated weight gain, hypertension, hyperglycaemia, and neuropathy, immunosuppression can have negative impacts on mental health and cause disordered sleep [23]. Additional MDT members could be trained to provide psychological support in the absence of a clinical psychologist. Immunologist support will be valuable to support management of immunosuppression where required. Hepatologist support will also be required in follow-up for guidance on safety, e.g. of alcohol use.

Intensive follow-up can impact PWH’s work commitments or educational opportunities. Support for patient liaison with employers may be valuable. Educational resources for employers and educators could explain follow-up commitments and implications of psychological effects.

## Next steps

Key areas that require further attention in preparation for haemophilia gene therapy include patient and HCP education, greater access to psychological support, and specific guidance for treatment implementation and follow-up. UK HCPs could engage with NHS commissioning bodies as a valuable starting point to guide discussions on requirements for implementation. This HCP engagement, and patient advocacy input into NHS commissioning decisions, should begin as soon as possible to ensure alignment with the community.

Ultimately, the patient journey is unlikely to progress in a linear manner. The decision to undergo gene therapy should not be rushed; alternative treatments for haemophilia that can maintain good health and a good quality of life should also be considered, and in-depth discussions balancing the potential risks/benefits are essential. The decision should be shared between PWH and HCPs and should move at each individual patient’s pace, ensuring that they and their families feel informed and fully supported throughout the process.

Comprehensive education should be an immediate priority for information providers, to ensure that PWH, their families, and HCPs are well prepared in advance of haemophilia gene therapy availability. Consideration of the challenges throughout the patient journey and unmet needs regarding gene therapy education and implementation in the UK will be integral to the future success of this promising therapy.

## Data Availability

Data sharing is not applicable to this article as no datasets were generated or analysed for this work.

## Abbreviations

EHC: European Haemophilia Consortium
EMA: European Medicines Agency
FDA: US Food and Drug Administration
HCP: Healthcare professional
MDT: Multidisciplinary team
NHS: National Health Service
NICE: National Institute for Health and Care Excellence
PWH: People with haemophilia
SMA: Spinal muscular atrophy
SPS: Specialist Pharmacy Service
UKHCDO: UK Haemophilia Centre Doctors’ Organisation
